# Designing for student autonomy combining theory and clinical practice – a qualitative study with a faculty perspective

**DOI:** 10.1186/s12909-024-05514-y

**Published:** 2024-05-14

**Authors:** Charlotte Silén, Katri Manninen, Angelica Fredholm

**Affiliations:** 1 Department of Learning, Informatics, Management and Ethics, Karolinska Institutet, Stockholm, Sweden; 2 Department of Infectious Diseases, Karolinska University Hospital, Stockholm, Sweden; 3 Centre for clinical research and education, County Council Värmland, Karlstad, Sweden

**Keywords:** Educational intervention, Implementation, Autonomy, Students, Faculty development, Clinical practice

## Abstract

**Background:**

Although extensive research exists about students’ clinical learning, there is a lack of translation and integration of this knowledge into clinical educational practice. As a result, improvements may not be implemented and thus contribute to students’ learning. The present study aimed to explore the nature of clinical faculty members’ learning related to how they apply research about student autonomy.

**Methods:**

A course, “Designing learning for students’ development of autonomy in clinical practice” was conducted for faculty responsible for students’ clinical education. Within the frame of the course the participants designed a project and planned how they would implement it in their clinical context. Fourteen clinical faculty members participated in the study. The participants’ interpretation of the educational intervention, which combines complex theory with the equally complex clinical practice, was explored by studying how the participants’ approaches and understanding of the facilitation of autonomy were manifested in their projects. The projects in the form of reports and oral presentations were analyzed using qualitative content analysis together with an abductive approach.

**Findings:**

One identified domain was “Characteristics of the design and content of the projects”. This domain was signified by two themes with different foci: *Preparing the soil for facilitating student autonomy*; and *Cultivating opportunities for students to actively strive for autonomy.* A second identified domain, **“**Embracing the meaning of facilitating autonomy**”** was connected to participants understanding of theories underlying how to support the development of autonomy. This domain contained two themes: *Connection between activities and autonomy is self-evident* and *Certain factors can explain and facilitate development of autonomy.*

**Conclusion:**

Education directed to strategic clinical faculty members to develop evidence-based approaches to student learning can be productive. To succeed there is a need to emphasize faculty members individual understanding of actual research as well as learning theories in general. Faculty trying to reinforce changes are dependent on their own mandate, the structure in the clinic, and recognition of their work in the clinical context. To achieve a potential continuity and sustainability of implemented changes the implementation processes must be anchored throughout the actual organization.

**Supplementary Information:**

The online version contains supplementary material available at 10.1186/s12909-024-05514-y.

## Background

Clinical education is a comprehensive part of health care education programs and therefore important and vital for health care students to become knowledgeable well-educated professionals. Research on students’ clinical learning has been extensively reported but there is still a lack of translation and integration of this knowledge into clinical educational practice. This is a problem when trying to improve clinical training and there is a need to understand more of this matter. Hence, this study explores clinical faculty members’ learning related to how they apply research.

### Introduction

The clinical environment offers a rich and powerful setting for learning and professional development [1–3]. In the clinical environment, abstract knowledge becomes tangible through its application in patient care. Students’ encounters with patients and staff representing their own and other professional groups provide unique experiences to reflect on and integrate in their learning. They can train and test their skills, observe, and examine patients, and provide care and treatment. Generalizable knowledge about diseases and their impact on people’s lives can be realized in each patient encounter. The variation that every patient encounter and personal experience offer enhances this knowledge. Learning in every encounter is not only related to the clinic as a physical place per se, but to relationships and experiences relating to this encounter [4–6]. As such, the clinic and the activities that occur in the clinic offer an inherent space for learning. This learning space provides contact with the reality of future professions, provides challenges, motivational factors, and feedback on behavior and thoughts. In this way, knowledge and professional development increases [2, 3, 7, 8].

Crucial aspects of creating and taking care of the rich opportunities for students’ learning are linked to the clinical environment, clinical supervisors and other stakeholders involved in the organization and implementation of clinical education. Responsible actors must engage, understand and be able to apply knowledge about how to support student learning to contribute to development. There is extensive research available regarding how to make the clinical learning environment fruitful for learning and to support students to reach their learning goals, while becoming well educated and well-functioning professionals (cf. [2–4], [8–11]).

However, several researchers point out that there is a lack of translation and integration of research-based knowledge into educational practice in the clinic [12–16]. This means that the main problem is not a lack of knowledge about student learning in the clinic, but the issue is why available research-based knowledge is not sufficiently applied. The complex nature of healthcare, the many faculty members involved, the lack of continuity related to education, and the fact that the education takes place in two different arenas – the university and the clinic – may explain some of the hindrances [14–17]. A major challenge in the development of health-care education is reaching and motivating faculty members to enhance their knowledge on how to improve student learning. This is especially true for faculty members involved in clinical education since their role mainly focuses on patient care and not on student education [12, 13]. There is an expressed need for research illuminating how further professional development for faculty members can lead to improvement of clinical education [12–16]. In particular, studies are needed to examine the connection between the individual clinical faculty member and their application of knowledge in the clinical context. A more in-depth understanding of these connections is paramount to support clinical faculty members to integrate research and thereby enhance student learning.

In the present study, an educational intervention directed to faculty responsible for students’ clinical education was designed, executed, and studied. The intervention sought to improve how clinical faculty understand and apply research regarding what may influence and stimulate student autonomy in clinical education. In connection with the educational intervention, a study was carried out that aimed to examine faculty learning in terms of integration of theoretical knowledge in the clinic. The findings were meant to contribute to the understanding of how to support clinical faculty members to apply educational research on autonomy and thus enhance learning and consequently professional development for students in the clinic.

### Autonomy and professional development

Development of autonomy is known to be crucial for student learning and professional development in the clinical setting. This concept was therefore chosen as the core of the intervention and this study. Nevertheless, autonomy is a complex concept, and its meaning is not possible to cover fully in the context of this study. The most fundamental aspects deemed important for this study will be presented below.

According to substantial research, autonomy is a fundamental need to experience self-governance and ownership of one’s actions [3, 18–23]. Development of autonomy in learning is the foundation of life-long learning, meaning the ability to move on, constantly reevaluate your own knowledge, ability to obtain and use information, and understanding of your learning processes [18, 19, 24]. Studies about promoting self-directed and/or self-regulated learning have shown the importance of taking into account factors such as student motivation, experience of control, ability to seek and apply knowledge, ability to discern learning needs and ability to evaluate the outcome of learning [18, 25–27]. Clinical practice needs autonomous health care providers, and here autonomy means something more than independence and control over your own learning. It has been shown that autonomy fosters personal identity and meaning, independent choices, responsibility, and critical thinking [19–23]. Important for professional competence is the ability to discern, assess, and pose new questions in unclear and incalculable situations [23, 24]. Research shows how a curriculum designed to strengthen autonomy can create a qualitatively different understanding of a subject or professional field, as demonstrated in student’s ability to link theory and practice with abstract thinking [22, 24].

In this study autonomy has been particularly connected to the meaning of authenticity and attachment. These concepts are identified as important parts of autonomy related to learning and professional development [2, 3, 22, 28, 29]. The rationale for this statement is outlined below. Autonomy is connected to authentic experiences in clinical training. It is also indicated that transformative learning processes that contribute to the development of a professional identity can be triggered by authentic experiences and the meaning-making of these processes [3, 28]. Manninen et al. [29] showed how authenticity in clinical education functions as a driving force for learning by creating meaning and relevance. Furthermore, Manninen [2, 29] has identified how authenticity can be both an external and an internal phenomenon, where external authenticity is produced by education and the surrounding environment – such as the interaction with patients in a clinical setting. Internal authenticity is experienced when students form mutual relationships with patients, feel a sense of belonging and perceive themselves as part of the team [2, 29]. Levett-Jones & Lathlean [30], stress the positive effects on learning that occur when students experience a sense of belonging in their clinical practice. These experiences of relationships and sense of belonging are captured in the concept of attachment and linked to the development of autonomy [3, 28]. Students need to be offered participation as well as actively strive to attach themselves to the actual clinical context in order to experience authenticity and autonomy in their learning. Prerequisites for students to experience and to seek attachment are based on mutual trust and respect [31, 32]. Several studies [3, 21, 22, 28, 29] showed how both autonomy and authenticity are social phenomena having to do with the relationships that students can form in their clinical education and the clinical relevance of given tasks. Thus, students can develop as autonomous professionals when they experience both external and internal authenticity. This includes opportunities to experience attachment and gain responsibility for relevant parts of patient care, as well as the opportunity to follow up on administered care [2, 3]. Students need to have access to and responsibility for entire processes, such as being able to evaluate the results of care and not just isolated actions or events. This reasoning applies to students, regardless of the clinical placement level since the complexity and length of processes can vary [33, 34].

### Designing for learning in the clinic

To further professional development, the design of learning in the clinic should offer students opportunities to experience through emotion and action what it means to be a professional nurse, doctor, or physiotherapist, etc [35]. By doing so, the risk for a narrow and static approach to knowledge decreases, thus making it easier to focus on knowledge application and the complexity of professional knowledge. A comprehensive review of the literature by Trede et al. [36] shows that the development of a professional identity is facilitated by learning based on cooperation and dialogue in practice and characterized by authentic experiences. Education should be designed to raise awareness of what autonomy means in clinical education to enhance student learning and the development of a professional identity. In turn this demands that clinical faculty members understand the concept and can integrate it in clinical education. The clinical application of evidence-based concepts means the ability to combine concepts and theory with a complex clinical practice [12, 14, 16, 17]. This is regarded as a challenge for faculty responsible for developing clinical education and supporting students in their learning.

### Aim

For the purpose of this study, an educational intervention was designed to present, explain, and illuminate theory and research related to supporting students’ development of autonomy. The study reached out to participants working in strategic positions in different clinical settings that enabled them to contribute to the design and development of clinical education in collaboration with different universities and educational programs. The participants designed and planned the implementation of a project aimed at enhancing student autonomy in their clinical context. *The aim of this study was to explore the nature of clinical faculty members’ learning related to how they apply research about student autonomy in their projects*.

## Method

The research approach was qualitative, interpreting participants’ experiences from a life-world perspective. The interpretation of meaning and lived experience was made possible through the tradition of phenomenological hermeneutics founded by Heidegger and further developed by Gadamer and Ricouer [37]. It is argued here that the lifeworld is mediated through narratives where individuals’ subjective understanding and sense-making of their lifeworld become visible [38]. Thus, the individual projects portrayed and studied here are viewed as narratives that manifest understanding of the phenomena under examination, the meaning of which is revealed through interpretation.

### Pedagogical framework for the intervention

The pedagogical framework described in the background regarding the development of autonomy and professional identity formed an important part of the content of the designed educational intervention and the present study. Additionally, the educational intervention was based on constructivist learning theories that emphasize active, creative processing of information, including cognitive, emotional, and social aspects as well as testing and practical actions (cf. [35, 39–42]). In the applied pedagogical framework, the lifeworld is seen as the total sum of the environment and everyday experiences that forms the individual’s world, thus forming the basis for the individual’s interpretations, thoughts, reactions, and actions [43]. Learning was seen as fundamentally situated in a physical as well as social and cultural context [39, 40, 43].

### Setting

The intervention, a course, “Designing learning for students’ development of autonomy in clinical practice”, was designed for health-care professionals responsible for students’ clinical practice in Stockholm County Council, or other participants with similar overarching clinical pedagogical work assignments. The relevant faculty role for this in Sweden is often an adjunct clinical lecturer (ACL) and this term will be used in the following description of the participants. They have their main employment and activities in the health care sector outside of a higher education setting and provide the university with specific expertise not found within the organization. The ACL supports both clinical supervisors and students at the clinical workplace, has the possibility to influence prerequisites for clinical education and functions as a bridge between the university and the local clinical education organization.

### The intervention – “Designing learning for students’ development of autonomy in clinical practice.”

The purpose of the course that constitutes the intervention in the present study was to strengthen the pedagogical competence of the ACL for her/him to understand the meaning of research-based knowledge about learning, and how to apply this knowledge in clinical supervision and teaching. The goal was that the ACL should be able to contribute to and support students’ opportunities to develop autonomy in learning. The intervention was designed aiming to help ACLs understand research about how to facilitate autonomy in clinical practice. The intervention design was built on the pedagogical framework described above. In one extensive and concluding learning activity, participants designed and implemented projects aiming to enhance student autonomy in their clinical context. These projects constitute the focus for analysis in this study.

The course was given online and included 5 weeks full-time study. The online design was believed to enhance accessibility and enable adaptation to individual clinical contexts. It was spread over 6 months to allow time for the participants to process the content of the course and to plan and implement their projects. The course consisted of both asynchronous parts and synchronous meetings using Zoom. However, the online design of the course and the analysis of outcomes related to this design is not within the scope of this study. Two of the authors, (CS, AF) were responsible for the course and acted as lectures and tutors. Other experts were invited to the synchronous meetings giving lectures and participating in discussions. A digital learning platform was created, and the participants were divided into groups of 4–5 participants and one tutor, who worked together mainly asynchronous online. The groups were mixed in terms of professional background and the nature of their clinical workplace to learn from each other and provide a range of perspectives while working with different learning activities. The content of the course was focused on the meaning of autonomy in learning and its application in clinical practice for students. The participants worked individually with written tasks and communicated with their group members and the tutor. They were asked to build on their previous knowledge and experiences and actively apply new knowledge and thoughts. The tutor facilitated communication in the group by posing questions and commenting on the written work and discussions. All learning activities were designed to allow participants to discern the relevance and implications of theory in their own individual clinical context and describe this with concrete examples. The core concepts of autonomy, authenticity and attachment were presented in lectures online and discussed synchronously. These lectures were also available on the digital platform.

In one extensive and concluding learning activity, participants designed and implemented projects aiming to enhance student autonomy in their clinical context. Participants worked on the project throughout the course, from a preliminary project plan to implementation, and evaluation. The projects were discussed in their groups as well as individually with the tutor. The projects were presented as written reports and final oral presentations synchronously in zoom. In the written reports, they described the design, theoretical background, implementation, and outcome of the projects. For the oral presentations, participants were asked to focus on what they perceived as most meaningful in their projects and how they applied pedagogical knowledge and reasoning.

### Participants

The course was open to all ACLs in the region. A written invitation to the course was spread through the regional network where ACLs are registered. A pedagogical course within higher education comprising at least 5 weeks full time study was required to take the course. The participants of the course were informed about the study and could volunteer to take part or not. There were 15 ACLs that took part in the course and fourteen of them participated in the study: seven registered nurses (five with postgraduate specialist nursing education), two radiology nurses, two biomedical analysts, one physician, one speech therapist and one occupational therapist. All participants were women, aged 36 to 63, with ACL experience from 1 to 13 years.

The context in which the study participants were active as ACLs mirrored the variations of the health-care field. Variations came to the fore related to in-patient and out-patient care, medical specialty and whether the unit offered a specialized service, such as a laboratory, radiology, or anesthesia at an operating department. The responsibilities and tasks of the ACLs were different. Some of them were responsible for students from one profession at various sites and others responsible for one unit and all students at that site. Others coordinated both supervisors and students within one unit, while others mainly acted as supervisors with a special assignment to act as an ACL at a specific site. The number of and kinds of students placed at the different units varied. In most cases, the ACLs were responsible for students from one profession and one educational level – undergraduate or postgraduate – but there were also examples involving several professions and different educational levels. The organization of the students’ clinical placements governed the ability for the ACL to plan activities. There were variations in the length of the placement and whether students stayed in one place or rotated between different departments. What could be designed to stimulate student autonomy depended on what the students were supposed to learn for their profession and on their educational level.

### Data collection

As described above the participants’ projects were chosen as objects for analysis. Data were collected using the written and oral accounts of the projects that constituted the concluding learning activity in the course. There were 11 projects included in the data. Three of them were collaborative projects where participants worked together; in two cases in the same clinic and specialty, and in one case from two different hospitals but in the same clinical specialty. Written accounts in the form of project reports were used together with video- and audio-recorded oral presentations of the projects.

### Data analysis

Based on the learning theories presented in the pedagogical framework for the intervention, the point of departure for the analysis was that ACLs showed what they had learned by planning and implementing projects in their own clinical setting with the aim of contributing to and supporting the students’ opportunities to develop autonomy. The application of the theory they had studied, i.e., the discernment of the meaning of the theory in the clinical context and in everyday practice, was made by the ACLs. Thus, it was the participants who expressed how they would use what they had learned to bridge the gap between theory and practice.

The participants’ learning was analyzed based on the written projects together with the oral presentation of these projects. An interpretative content analysis of both the manifest and latent content was performed [44, 45]. The manifest content refers to data close to the expressions used by the participants, in this case the written and oral descriptions of the projects. The latent content refers to the authors’ interpretation of the meaning of what is expressed related to the development of autonomy. An abductive approach was also applied, and thus data were analysed iteratively going back and forth between parts and wholes, both inductively, and deductively informed of theoretical perspectives during the research process [46]. The theoretical foundation for analysis was the above-described concepts furthering the development of autonomy. The inductive part of the analysis aimed to contribute to new perspectives and a development of how these concepts can be interpreted.

Condensed meaning units were extracted in the written projects and the video- and audio- recordings and subsequently coded. The codes were compared for differences and similarities and grouped into categories describing variations in focus and approaches to support development of student autonomy. In the next step, categories were interpreted and designated as latent themes. The identification of manifest and latent content of the participants understanding of theories underpinning autonomy, was built on revisiting the condensed meaning units and codes with focus on critical features, the relationship between concepts and practice, and how concepts were connected [44, 45]. Thus, one project can be represented in both themes and several of the categories. Examples illustrating the steps of the data analysis process are described in Appendix 1. Two of the authors (CS, AF) performed the basis of the analysis iteratively independently and together. Emphasis was put on reflexivity concerning preconceived interpretations related to the researchers’ involvement in the course. The preliminary findings were critically reviewed by the third author (KM), less involved in the course, and then discussed and negotiated between all the authors to achieve consensus. All authors have extensive knowledge and experiences of clinical education as teachers, clinical supervisors, and as experienced qualitative researchers in medical education. The researchers’ collaborative analysis was meant to contribute richness and credibility to the findings.

## Findings

**Fig. 1:**
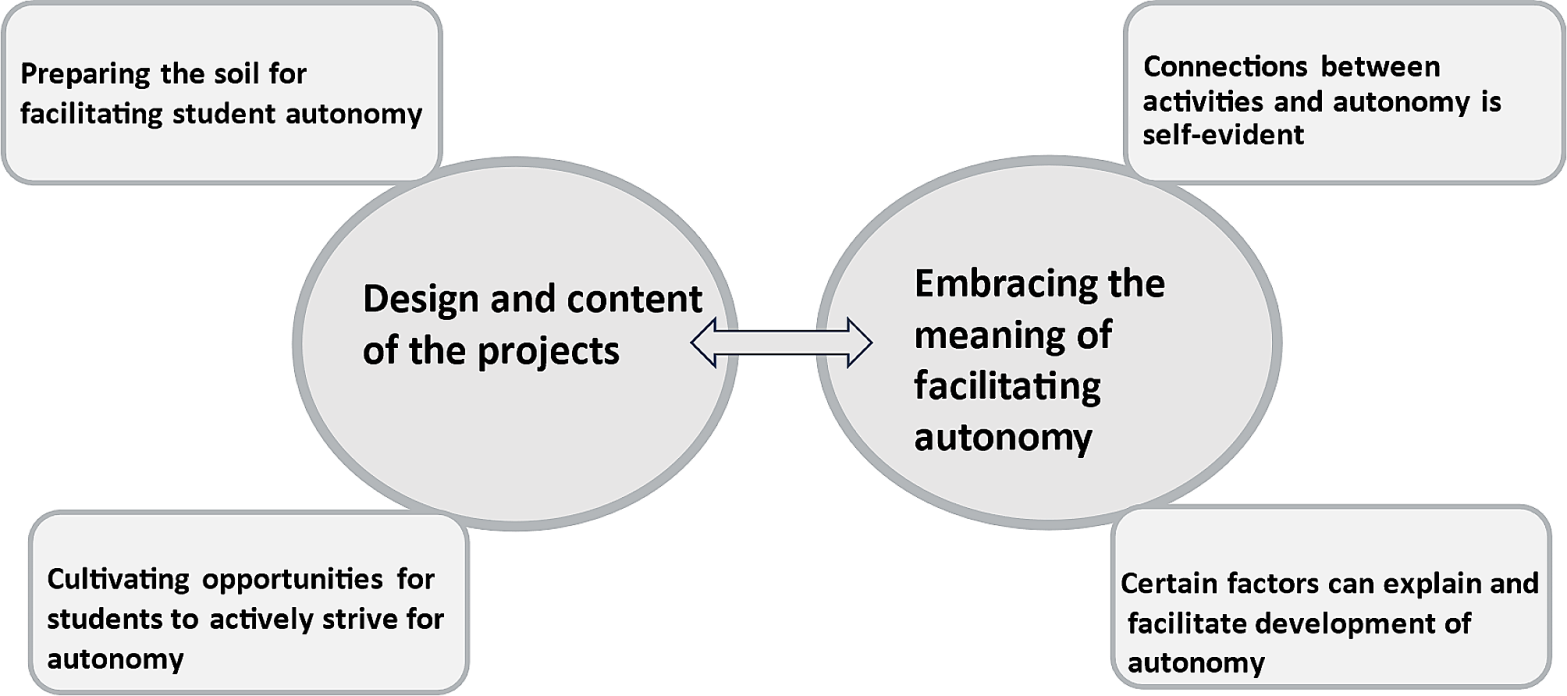
- Overview of findings

The analysis of the content and implementation of the participants’ projects is described in two domains. **A: Characteristics of the design and content of the projects, and B: Embracing the meaning of facilitating autonomy.** Domain A was related to the description of the projects based on who was engaged and the focus of the content of the implementation. The categories and themes related to domain A illustrate the outcome of learning in terms of how they organize activities to implement student autonomy. The basis for the categories and themes in domain B was the analysis of the meaning of the projects and how the participants talked about how to achieve autonomy. The findings in Domain B relate to the participants’ learning in terms of their understanding of how to apply the theories underlying the support for the development of autonomy. An overview of the findings is displayed in Fig. 1. Quotes are presented with numbers of the participants and marked with *oral account* (oa) and *written account* (wa).

### Domain A: Characteristics of the design and content of the projects

The way the participants designed and described their projects on how to support students’ development of autonomy in clinical practice varied. **Two themes with different foci were identified; Preparing the soil for facilitating student autonomy; and Cultivating opportunities for students to actively strive for autonomy**. The first theme comprised two categories; *Engaging supervisors to support student autonomy* and *Emphasizing organizational dimensions that have an impact on implementation.* The second theme was characterized by the two categories *Activities involving students during significant parts of their clinical placement* and *Specific activities focused on certain knowledge and skills.* The content of Domain A is illustrated in Table 1.

**Table 1:** Domain A: characteristics of the design and content of the projects

Characteristics of the design and content of the projects	
**Themes**	**Categories**
Preparing the soil for facilitating student autonomy	*Engaging supervisors to support student autonomy* *Emphasizing organizational dimensions that have an impact on implementation*
Cultivating opportunities for students to actively strive for autonomy	*Activities involving students during significant parts of their clinical placement* *Specific activities focused on certain knowledge and skills.*

#### Preparing the soil for facilitating student autonomy

Several participants chose to **prepare the soil**, that is, they utilized their knowledge of developing student autonomy to prepare the clinical practice environment for the students. Within this theme two categories were identified: *Engaging supervisors to support student autonomy* and *Emphasizing organizational dimensions that have an impact on implementation.* The first category focused on the supervisors, and the second took a broader approach, including the structural factors of the clinical placement, the supervisors’ role, and managers on different levels.

##### Engaging supervisors to support student autonomy

To reach the goal of fostering independence in students, the supervisors in the clinic were engaged by the ACLs. One approach within this category was to focus on support for supervisors by offering written plans, advice and thoughts on factors that have an impact on student independence in the clinic.

The documents written by the ACL varied between concise information sheets, and comprehensive plans on activities. The documents contained hints and advice about how supervisors should act to facilitate student independence. The supervisors’ responsibilities in relation to the students were also pointed out. A document could be created by the ACL, presented, and handed over to the supervisors to use. Other documents were authored by the ACL, presented, discussed, and adapted to the supervisors’ comments before they were finalized and used.

*“In the meeting with the supervisors, we used time to discuss the guide (advice to the supervisors to support student autonomy). Many supervisors attended the meeting …we believe we have created a great participation”* (No 12 and 13, oa).

Another example was a comprehensive operational plan aiming to develop a pedagogical framework for supervision that would increase the supervisors’ own confidence, allowing the students to practice autonomy in their professional role.

*“The plan is that the supervisor guides the student from Active Observations, involves the student to Work Together, then invites the student to Take a Lead and finally Work Independently “*(No 1, wa).

The ACLs instructions to the supervisors differed. In some cases, the content of the documents was left for the supervisors to use as they saw fit, while other documents contained prompts on how to use it and how to act.

Follow ups of the content of the created documents and usefulness of the initiated guidance for the supervisors varied. In some cases, there were no systematic plans for follow up, while others discussed and revised the content of the document.

Another approach within the category of preparing the soil was signified by activities that process the meaning of and facilitation of autonomy. The aim of these activities was to stimulate reflection on factors affecting the development of autonomy to ensure continuation.

*“To make the implementation* [new activities] *work, there is a need for careful and long-term planning ….it takes even more communication* [*between different stakeholders*] *…and it must be adapted to the students’ actual clinical placement”* (No 8 and No 14, wa).

An activity taking the form of a workshop was characterized by the ACLs presenting what autonomy and factors stimulating student development might mean for the students.

*“I created a workshop and gave a lecture about the concepts we had studied in the course – about how to facilitate autonomy…. I talked about attachment, trust, and authenticity and how that relates to autonomy”* (No 10, oa).

One activity consisted of recorded lectures by the ACL combined with prepared tasks for the supervisors to complete. In the lectures, the different factors presented in the course that the ACLs had attended were presented. Through the tasks, the supervisors were encouraged to reflect on what these factors might mean for their students in their own clinical context. It was up to the supervisors to decide when they wanted to study the recorded material.

*“The recorded lectures combined with tasks were meant to provide those responsible for the students in clinical practice knowledge about factors influencing students’ possibilities to reach autonomy during their placement……It would provide them with tools and ways of thinking to be able to change the organization of the clinical practice towards the goal of increased autonomy for students”* (No 11, wa).

##### Emphasizing organizational dimensions that have an impact on implementation

In this category, the importance of considering organizational structures of both the health-care unit and the students’ clinical education when implementing measures to stimulate student autonomy were pointed out. The dimensions brought up and considered in the participants’ projects were the structure of the clinical practice, such as how responsibility of supervision was distributed, and the total number of students at the unit, but also the spread of students over a semester. The ACLs considerations included the duration of the placement, how and whether the students were rotating between different sections at the unit or stayed in one place. To enhance development of student autonomy, the idea of continuity in supervision was emphasized, and to maintain sustainability, managers on several levels were engaged in the planning, as were supervisors.

*” We involved managers and administrative assistants in our planning…. administrative assistants plan supervisors’ work schedules and thus influence their work…. a pedagogical encounter was set up to engage them* [*managers and administrative assistants*] *in a pedagogical discussion…. We also created a group with supervisors from different departments of the clinic to discuss the project and the purpose of the idea of achieving continuity”* (No 8 and No 14, wa).

Communication and cooperation with the university was stressed as crucial for whether the supervisors succeeded in facilitating student autonomy. There was an agreement among the ACLs in this category on the importance of including managers from different levels to succeed with the planned project.

*” We contacted our manager and presented the project to her, and we contacted our administrative assistants and informed them about who the main supervisors were so that their shift work would not be affected”* (No 4, oa).

#### Cultivating opportunities for students to actively strive for autonomy

In this theme, **cultivating opportunities for students**, another significant approach to use the knowledge of autonomy was identified. The ACLs planned activities directly to students to stimulate their development of autonomy. The theme consists of two categories: *Activities involving students during significant parts of their clinical placement* and *Specific activities focused on certain knowledge and skills.*

##### Activities involving students during significant parts of their clinical placement

Activities in this category were planned to capture multiple competences and were integrated throughout most of the practice period. A characteristic activity planned by the ACLs included in this category involved assignments that were identified and described for the specific students to complete in pairs.

*“Learning activity: The students listen and observe a professional encounter with a patient. Afterwards they attend a workshop about how to document data in a patient’s chart…. The students work together and give each other feedback …. they are asked to go on working like this, writing on their own, discussing with each other and after that consulting the supervisor”* (No 10, oa).

The planned activities were related to students’ vocational training and involved ideas about progression regarding students’ possibilities to act autonomously and the complexity of the assignment itself. Students trained different skills on their own and did not only watch their supervisors. They had to make choices between different actions, such as how to proceed in a certain situation, as well as judge when an assignment was finished and how it should be reported. Similar activities were also planned for other students without emphasis on peer learning. Instead, the supervisor continuously identified learning tasks for one student at a time, and thus independence was gradually required.

Variations could be noted in the ACLs approach to stimulating autonomy in these activities. In some cases, the learning tasks were planned in detail by the ACL and the training was limited to certain skills and behaviors. Other approaches were planned to continuously encourage the student to take responsibility and perform independently.

*…the supervisors were encouraged to give students increased responsibility, e.g. by allowing them to be supervised by colleagues/other professions/other students and receive more assignments to solve themselves…. such as that they can develop a sense of autonomy in parallel with a sense of belonging with the whole health-care team and the workplace.”* (No 10, wa).

Students’ reflections on their own performance were emphasized as important to stimulating development of autonomy. Sometimes, the reflection sessions were mainly about how the tasks had been carried out. In other cases, the students’ own perceptions about their progression towards autonomy were also important to discuss.

*“The ACL met the student and the supervisor every week to reflect, based on a certain model. The core concepts of autonomy, authenticity, attachment, trust, and professional identity were discussed to evaluate whether the students felt that they experienced autonomy at the clinical placement. The students were asked to write in their logbook and their questions were discussed during the weekly reflection time.”* (No 7, wa).

##### Specific activities focused on certain knowledge and skills

In this category, ACL’s planned projects contained learning tasks that focused on single skills completed at a specific time during the placement. The aim was to stimulate the development of autonomy in different ways connected to this learning task. A typical kind of activity was characterized by the ACL creating conditions for the students’ training but leaving the implementation to the students. These kinds of activities could be about connecting well-planned written tasks to common clinical issues for the patients who were cared for on the ward. The students could choose when to review the written tasks and how to perform them.

*” It is difficult for students to feel attachment and ‘to be nurses’ on the ward when they are there for a short period of time, the patients are very ill, and they don’t have the right knowledge and skills to independently take care of them. I created written learning activities for students to work with on their own or together with other students…it meant that they could select a patient to talk to, search for knowledge and consider questions about a patient’s status and appropriate care”* (No 5, oa).

Another activity took the form of a room prepared with equipment, offering opportunities for students to independently train important professional skills. The aim of this activity was to facilitate autonomy and critical thinking within postgraduate nursing specialist training.

*“The students can practice together to supervise the monitoring and treatment of a simulated patient based on an authentic scenario. This means that they themselves lead the activity and must make important decisions and reflect on the outcome after a presentation of a project* (No 2, oa).

Most activities planned by the ACLs focused on one profession at a time but there was one example targeting interprofessional learning. Interprofessional seminars were implemented for students to learn about other professions from their peers, and these seminars were followed up with reflections on what professional teamwork meant for the development of professional autonomy.

### Domain B: Embracing the meaning of facilitating autonomy

The participants’ choices of design and the ways they described and talked about their projects also reflected a dimension of their learning related to their theoretical understanding of development of students’ autonomy. The analysis of the participants’ descriptions of how different factors facilitate and relate to the development of autonomy resulted in the outcome of two qualitatively different perceptions, here designated as two themes: **Connection between activities and autonomy is self-evident** and **Certain factors can explain and facilitate development of autonomy.** The first theme consists of one category: *Lack of reasoning about the meaning of autonomy*. In the second theme, two categories emerged: *The concept of autonomy as a core value* and *Various factors are linked to the development of autonomy.* The content of the domain is illustrated in Table 2.

**Table 2:** Domain B: Embracing the meaning of facilitating autonomy

Embracing the meaning of facilitating autonomy	
**Themes**	**Categories**
**Connection between activities and autonomy is self-evident**	*Lack of reasoning about the meaning of autonomy*
**Certain factors can explain and facilitate development of autonomy**	*The concept of autonomy as a core value* *Various factors are linked to development of autonomy.*

#### Connection between activities and autonomy is self-evident

One category denoted this theme, namely the lack of reasoning. The activities were described as facilitating autonomy, but there was no explanation for the underlying ideas of *why* the activities facilitated autonomy. One example is that a project was meant to introduce peer learning and activities for the students were thus described. These activities could involve students training skills on their own and they were asked to make their own decisions and discuss with their peers. However, there was no elaboration on how and why these activities were supposed to result in students becoming more autonomous. The relationship between activities and the ability to make choices and more independent decisions seemed to be taken for granted. Another example is the notion that activities directed at interprofessional education led to autonomy, which also was never explained.

*“They develop autonomy as they see their own responsibilities as they are reflected in what other professions perform and are responsible for. It promotes their own professional development.”* (No 9, wa).

#### Certain factors can explain and facilitate development of autonomy

The main qualitative difference between this theme compared to the first is that the participants explained and reasoned about how and why certain activities stimulated and led to independence. However, which factors that were brought up varied, as did the complexity of related explanations and reasoning.

##### The concept of autonomy as a core value

The level of understanding in this category was characterized by explanations and reasoning linked to the use of autonomy as an overarching concept. Here, an activity such as being asked to independently use a skill or to handle an encounter with a patient was chosen because this training would lead to student autonomy. When the project was introduced to supervisors or managers, the planned activities were mainly motivated by claiming that if students were given opportunities to act independently, it would foster autonomy in them. Factors brought up in the course as influencing development of autonomy were not used by the ACLs to elaborate on how to facilitate autonomy. They didn’t elaborate on any other factors brought up in the course as influencing the development of autonomy.

*“When we thought about how to work with student autonomy, we decided to use peer learning. The students’ assignments are described, they work together, and the students take responsibility to carry them out. The students can stand on their own two feet… they are trusted”* (No 6, oa).

##### Various factors are linked to development of autonomy

This category was characterized by an elaborate understanding of the meaning of autonomy and factors that have an impact on the development of autonomy. The ACLs reasoning about autonomy and other factors influencing autonomy was complex to a varying degree. Some participants explained and related their activities to one or more factors.

*“…the students meet new supervisors very often, generally speaking every day… this leads to obstacles for student learning, and it makes it difficult to develop autonomy and authenticity. Both the students and the supervisors become ambivalent when they must create new relationships almost every day”* (No 8 and No 14, wa).

Others reasoned about how different factors were interdependent and related to facilitation of autonomy in a broader sense.

From a guide for supervisors: …*we think this is about attachment, the students are invited, and they have got a place when they arrive….and this next guiding advice is connected to trust… we trust the student that they know a lot, but it takes time to learn this new specialty. It is about autonomy too – that the students take responsibility and think for themselves… Some supervisors are very controlling, so the students don’t have the possibility to practice how to really be critical care nurses, so they don’t experience authenticity”* (No 12 and No 13, oa).

## Discussion

We argued in the background that a hindrance for development of a rich learning environment in clinical education is that available research-based knowledge is not sufficiently applied [12–17]. A way to face this problem is to enhance knowledge about how clinical faculty members understand and integrate theoretical knowledge in clinical practice. In this study we examined projects designed and implemented by clinical faculty members to find out how they, in this case, applied research about student autonomy in clinical education. The projects were the final part of a course introducing theories and research on the importance of students’ development of autonomy in clinical education. The purpose of describing and reasoning about these participants’ learning was to contribute to a deeper understanding of how to support clinical faculty to acquire and apply theoretical knowledge in clinical practice.

Two different domains mirroring the participants’ learning outcome were identified when their projects were analyzed. One domain concerned what they had decided to focus on to facilitate students’ development of autonomy and how they went about implementing their ideas in the clinic. The other identified domain involved the interpretation of the participants’ understanding of the theoretical framework underpinning autonomy as a concept. These findings expose different perspectives on how a course with a specific design directed at faculty members impacted their actions and understanding, i.e., the faculty members’ learning. The outcome of the course was encouraging in relation to the facilitation of student learning in clinical practice. All the participating ACLs projects contained activities and/or documents that involved some form of application of theories on how to facilitate student autonomy Some projects aimed to “prepare the soil”, such as educating supervisors and creating fertile ground for learning for the students. Another group of projects were planned directly for students, signified by “cultivating opportunities**”** for them to practice autonomy through certain activities. Several studies have shown that the nature of clinical education is complex [12, 14, 16, 17]. The students’ education takes place in two different arenas – the university and the clinic. Many faculty members are involved in clinical education, and their role mainly focuses on patient care and not on student education [12, 13]. It became obvious that this complexity of clinical education influenced what the ACLs assessed possible to accomplish. This was mirrored in the choices that the ACLs made concerning the content and to whom they directed their projects. A comprehensive review of research on student learning in clinical practice found that issues about how to organize students’ learning were the most researched, indicating that organizational issues are an essential part of change [47]. The significant features of the health-care units in which the ACLs acted had a large impact on how they planned and implemented their projects. This underlines the importance of being familiar with the nature of context to introduce changes. The projects that were targeted supervisors, managers, and the organization presumably had an impact on a wider group of students compared to projects that were designed directly for a minor group of students or supervisors. This is important in relation to issues about sustainability. If faculty members on different levels in the clinic are engaged, ideas and knowledge about how to facilitate student learning can continue to develop and gain a foothold [14, 48–50]. Projects involving managers and system levels are far more likely to become sustainable [13, 15, 17, 48]. The activities planned directly for students, and where the ownership of the ideas was closely linked to the ACLs, run the risk of being dependent on a limited group of faculty members, and may cease as soon as the person in charge is not there.

The variety of planned activities and documents created by the ACLs displayed comprehensive understanding, challenges and shortcomings related to the meaning of autonomy. Two qualitatively different levels of understanding of autonomy and how different factors facilitate and relate to the development of autonomy emerged [3, 22, 24]. One level of understanding relied on a presumed self-evident relationship between an activity and student autonomy. The other level of understanding involved explanations for how different factors, such as authenticity, trust, and belonging, relate to the development of autonomy. These differences in learning outcomes are very important to consider in faculty development. We claim that a level of understanding that includes the ability to discern the meaning of theory in the clinical context and in everyday practice, is necessary to support others, such as supervisors and students, and implement sustainable change. The understanding of a situation and the understanding of the phenomenon that gives this situation meaning are connected. According to Marton and Booth [24] a situation is understood based on the phenomena involved – and the phenomena are perceived in light of the specific situation. When viewed in relation to the participants’ projects this meant that the understanding of the theory could be seen in the choices the participants made regarding their projects, what they perceived as important issues, how they proposed to solve these issues, etc. From a variation theory perspective, this is viewed as a matter of discrimination and differentiation, and learning is seen as the ability to discern these differences [24, 35]. The space for learning, therefore, is the potential variation or difference provided by the situation [24, 35]. Opportunities to participate in continuing professional courses and forums for discussions between ACLs may support the development of a deeper understanding of theory when it is linked to clinical practice.

In the design of the course emphasis was placed on participants creating and implementing a project. What did that mean for their learning? Some projects were quite limited, as they sometimes only comprised one document, or when an activity only reached a small group of students or a minor group of supervisors. This can be a shortcoming, but it is possible that further development and successful implementation is more dependent on the properties of the document or activity related to theoretical understanding and the ACLs ability to identify meaningful problems. If an initiative is well substantiated, there will be more opportunities to build on it [35, 43]. Some projects were broader, where several activities were planned to be repeated and continued over time and they engaged both supervisors and students. These projects carry a high potential for successful implementation and impact on supporting student learning, since continuity and engagement increases opportunities for faculty members to learn [17, 35, 50].

Above, we have discussed how the ACLs’ learning manifested itself in the participants’ projects and how that reflected their understanding of theories about learning processes, connected to development of autonomy. The group of ACLs is particularly interesting as they support both supervisors and students and are responsible for bridging the education gap between the university and clinical practice. Understanding of the actual subject matter – in this case facilitating autonomy – turns out to be very important for the individual ACL to manage to drive development and change. It impacted problems that they discerned and identified, and the choices they made in their planning [24, 35]. Successful implementation also seems to depend on the mandate of the change agents, in this case the ACLs and their awareness of the practices at their unit and on different management levels [13, 17, 48–50]. In addition to these requirements, we would argue that understanding how people learn in general is also critical to the implementation of new ideas. This statement is based on the application of the constructivist pedagogical framework underpinning this study [35, 39–43]. If development is to occur, all stakeholders must process and understand the meaning of autonomy and be able to relate and link it to their practice [13, 17, 48–50]. The way the ACL communicated with and involved affected parties in their projects revealed their awareness of learning processes not only directed at facilitating autonomy. There were examples of projects in which an activity and/or a document was created by the ACL and the main strategy during implementation was to provide information about it to supervisors, managers, and students. This strategy essentially meant that no learning processes were initiated to facilitate understanding. In other projects, supervisors and/or students were involved to different extents in creating and making their own choices about how to perform suggested activities and review documents. These strategies encouraged the stakeholders to think about and react to practice, as well as reflect on what autonomy meant to them personally. Other factors conducive to active learning processes that were identified were planned follow-up opportunities, interactive feedback, and shared recurrent encounters to discuss documents and/or activities.

### Strengths and limitations

The strength of this study lies in the theoretical and conceptual rigor applied throughout both the design process and the implementation of the educational intervention. There is also considerable procedural rigor due to the intervention being implemented with particularity and a firm epistemological stance. Limitations are connected to the sample of the study with only interested and ambitious learners who decided to take this course. However, this also contributes to rich data descriptions. There are notable challenges in studying an intervention that we as researchers have designed and the outcomes of this intervention. These challenges have been counteracted through constant reflexive discussions and questioning of assumptions.

## Conclusion

This study shows that an educational intervention that emphasizes application of theoretical knowledge in clinical practice can enhance the development of evidence-based approaches to support students’ learning. Targeting a strategic group, such as the ACLs in this study, can be a successful way to strengthen faculty development. All participants in the intervention demonstrated the ability to use theoretical knowledge and create activities to support students’ learning. However, their applications differed in terms of underlying reasoning, reach and potential sustainability. To some extent these differences were due to a deeper understanding versus a more superficial understanding of the central concepts related to autonomy. Another critical factor affecting implementation was the ACLs understanding of learning processes in general. Lessons to learn for professional faculty development are that there is a need to stress individual understanding of actual theoretical concepts as well as learning theories in education addressing clinical faculty. The outcome of the ACLs planned projects turned out to be very dependent on their own mandate, the structure in the clinic, and acknowledgement of their work in the clinical context. This study also highlights that in order to achieve a potential continuity and sustainability of implemented changes in the clinic the implementation processes must be anchored throughout the actual organisation.

### Electronic supplementary material

Below is the link to the electronic supplementary material.

Supplementary Material 1

## Data Availability

The datasets generated and analyzed during the current study are not publicly available due to ethical reasons connected to the participant’s informed consent. The data generated during this study consists of written reports and transcribed audio recordings of participants who have been guaranteed confidential handling of data. On reasonable request, data can be made available from the corresponding author.
