# Supplementary material for: Designing for student autonomy combining theory and clinical practice – a qualitative study with a faculty perspective
Source: BMC Med Educ. 2024 May 14;24:532. doi: 10.1186/s12909-024-05514-y (PMC11092088; doi:10.1186/s12909-024-05514-y)
Supplement: Supplementary file 1 — Supplementary Material 1 [file 12909_2024_5514_MOESM1_ESM.docx]

Appendix 1: Examples illustrating the steps in the data analysis process

**Domain A Characteristics of the design and content of the projects**

| Meaning units | Codes | Categories | Themes |
| --- | --- | --- | --- |
| We have created a guide for the supervisors to highlight how they can encourage students to work more independently. | Activities directed to supervisors | *Engaging supervisors to support student autonomy* | **Preparing the soil for facilitating student autonomy** |
| I wanted to improve the supervisors' conditions, which in turn created the conditions for the students' independence. I therefore chose to target them and the organization of the student reception. | Strategies aimed at supervisors, management, administrators | *Emphasizing organizational dimensions that have an impact on implementation* |  |
| Peer Learning must be used during clinical practice. The students may take responsibility for planning and implementation of certain tasks together. | Plans directed to students during their placement | *Activities involving students during significant parts of their clinical placement* | **Cultivating opportunities for students to actively strive for autonomy** |
| Students from different professions work together on a patient case to understand how they can develop autonomy from their professional responsibilities. | Focus on creating one activity | *Specific activities focused on certain knowledge and skills.* |  |

**Domain B: Embracing the meaning of facilitating autonomy**

| The students work with the given tasks on their own. | No explanation to how and why this can lead to autonomy | *Lack of reasoning about the meaning of autonomy* | **Connection between activities and autonomy is self-evident** |
| --- | --- | --- | --- |
| To promote students’ autonomy, we have chosen to clarify the role of the main supervisor. | All clarifications relate mainly to the concept autonomy | *The concept of autonomy as a core value* | **Certain factors can explain and facilitate development of autonomy** |
| It is important for the supervisors' experience of authenticity that they benefit concretely from the seminar series and that it is comprehensible, so that the theoretical knowledge can be used in daily student work. They must be given tools to understand for themselves how they can influence the students' opportunity for connection and trust, authenticity, and independence. | Core concepts are mentioned.  Relationship between the concepts and learning | *Various factors are linked to development of autonomy* |  |
